# Individual-level socioeconomic status and cataract-induced visual disability among older adults in China: the overview and urban-rural difference

**DOI:** 10.3389/fpubh.2024.1289188

**Published:** 2024-01-18

**Authors:** Yunyi Fan, Shuai Guo, Wanwei Dai, Chen Chen, Chun Zhang, Xiaoying Zheng

**Affiliations:** ^1^HeSAY/Institute of Population Research, Peking University, Beijing, China; ^2^School of Population Medicine and Public Health, Chinese Academy of Medical Sciences & Peking Union Medical College, Beijing, China; ^3^Department of Ophthalmology, Peking University Third Hospital, Beijing, China; ^4^Beijing Key Laboratory of Restoration of Damaged Ocular Nerve, Peking University Third Hospital, Beijing, China

**Keywords:** cataract-induced visual disability, socioeconomic status, older people, rural-urban inequality, China

## Abstract

**Objective:**

To investigate the prevalence of cataract-induced visual disability and its association with individual-level socioeconomic status (SES) among older adults in China.

**Methods:**

Using the data of 354,743 older adults (60 years and older) from the Second China National Sample Survey on Disability in 2006. Cross-sectional study design was applied. The differences in visual disability prevalence of cataracts among sociodemographic subgroups were analyzed by the chi-square test, and the association between individual-level SES and cataract-induced visual disability was investigated by the multivariate logistic regression model.

**Results:**

The weighted visual disability prevalence of cataracts was 4.84% in 2006. Older people with a higher household income *per capita* (OR = 0.83, 95% CI: 0.81–0.85), higher education level (primary school vs. illiteracy: OR = 0.80, 95% CI: 0.76–0.83; ≥undergraduate college vs. illiteracy: OR = 0.31, 95% CI: 0.25–0.39), and occupation (OR = 0.53, 95% CI: 0.50–0.56) were less likely to suffer from cataract-induced visual disability. Household income *per capita* and education level increase played a greater role in decreasing the risk of visual disability caused by cataracts in urban areas, while having occupation contributed more to reducing the risk of disability in rural areas.

**Conclusion:**

The gap in individual-level SES is closely related to the visual health inequities among older Chinese people and there are two distinct mechanisms in rural and urban areas. Strategies to promote collaborative healthcare development regionally, strengthen safeguards for disadvantaged groups, and increase public awareness of visual disability prevention are warranted.

## Introduction

Cataracts, the leading cause of reversible blindness and visual impairment globally (1, 2), also ranked the first cause of visual disability in China (3, 4), have become an important public health issue and brought significant individual and social costs worldwide (5, 6). A cataract is a lens abnormality characterized by decreased transparency and increased cloudiness (2). Given that it worsens over time, people left untreated will endure increasingly severe vision impairment, which can lead to disabilities, even blindness (1). Studies have demonstrated that visual disability will bring adverse physical and psychological consequences to the disabled and their caregivers (1, 7–12), such as difficulties with activities of daily living, lower quality of life, higher risk of comorbidity of dementia, and so on. According to the World report on vision and the Global Burden of Disease Study, vision impairment has posed an enormous global financial burden (1). In China, years lived with disability for vision impairment have increased by 69.4% from 1990 to 2015 (13). As China’s population rapidly ages, the scale of disabilities caused by cataracts is projected to soar in the coming decades (14), considering that age-related degeneration causes most cataracts (2). Meanwhile, population growth, changes in behavior and lifestyle, and ongoing urbanization will aggravate the crisis (1). By 2050, experts project that about 240 million middle-aged and older people in China will be affected by any cataract, while around 187 million will have age-related cataracts.

Improving people’s eye health is one of the prime targets of health policy in China. As a preventable and treatable visual disease, the concern and intervention of cataracts will reap significant benefits for public health and socio-economic development. In consideration of reducing cataract-induced visual disability, the context in which risks are produced needs to be better understood. Some studies explored the factors influencing visual disability worldwide, including sex, age, and some socioeconomic factors (15–17). However, compared with the epidemiological prevalence studies, few research has focused on risk factors of vision disability in China, and studies based on national-level data are lacking. Most of the limited literature focused on medical and pathological aspects, sociodemographic perspective received little attention, especially regarding socioeconomic status (SES). Although the national prevalence of age-related cataracts has reached up to 73% among Chinese adults aged 85–89 (1), more studies investigated visual health in a wide age range or preschool children (18). Furthermore, China still faces the long-standing rural–urban dual structure, even considering the deepening of healthcare and medical system reform. Relevant studies have shown that disabilities, including blindness, are more common in rural areas (15, 19), while visual impairment has a greater impact on the well-being of urban residents (12). The necessity for us to distinguish different mechanisms between urban and rural areas was indicated.

Based on a national, population-based dataset from the Second China National Sample Survey on Disability (CNSSD), this study aimed to estimate the prevalence of cataract-induced visual disability among older adults in China. The first CNSSD was conducted in 1987, and the Chinese government plans to investigate every 20 years. Therefore, the data used in the present study is the most currently available information. We further evaluated the association between SES and cataract-induced visual disability and whether there are distinct patterns between urban and rural areas. This work is necessary for identifying the priority population and areas for preventing vision impairment, which is essential to the National Eye Health Program and the WHO global eye care targets for 2030.

## Methods

### Study design

Cross-sectional study design was applied. The CNSSD was implemented from 1 April to 31 May 2006 (20), which investigated the prevalence, causes, distribution, and severity of disabilities in China (20). A multistage, stratified random-cluster sampling strategy was applied to select 2,526,145 non-institutionalized individuals from 31 provincial-level regions in Mainland China, which have a national representative of the Chinese population (20). Among each division, sampling strata were defined according to subordinate administrative areas, local demographic indicators, local geographical characteristics, and local socioeconomic development to allow for anticipated regional variability and reduce the sampling error (21). A probability proportional to the cluster size method was used within each stratum. After counties were randomly selected from provincial-level administrative divisions, towns were randomly selected from counties. Villages/districts from towns and finally communities from villages/districts (21). All households in selected communities were investigated (20). 734 counties, 2,980 towns, 5,964 survey communities, and 771,797 households were selected. The participation rate was 99.8% (20). The survey is the most recent nationally representative survey on disability in China (3, 4, 22–24).

The investigation was conducted in two stages. A combination of household surveys and professional medical examinations was used to collect data. Firstly, the trained investigator, accompanied by assistants who are familiar with the communities, visited the household and inquired about the family members aged 7 years or older to collect the demographic information and screen for suspected visual, hearing, speech, physical, intellectual, and mental disabilities based on the structured screening table of the CNSSD (21). For those aged 0–6 years, investigators filled in the children’s health examination registration form. Secondly, communities set up stations for designated specialists to do further disability examination intensively. Those 7 years and above suspected disabled received medical tests and were graded according to the Grading Standard of Disabilities of the CNSSD (21). For children aged 0–6 years old, experienced doctors conducted disability screening, diagnosis, and confirmation (21). Finally, 161,479 disabled persons were identified (20). 738 survey teams, more than 20,000 investigators, 6,000 doctors of various, 730 statisticians, and 50,000 survey assistants attended this survey (20). Experts at home and abroad repeatedly demonstrated the quality of the survey data, which was considered reliable.

### Study sample

**Figure 1:**
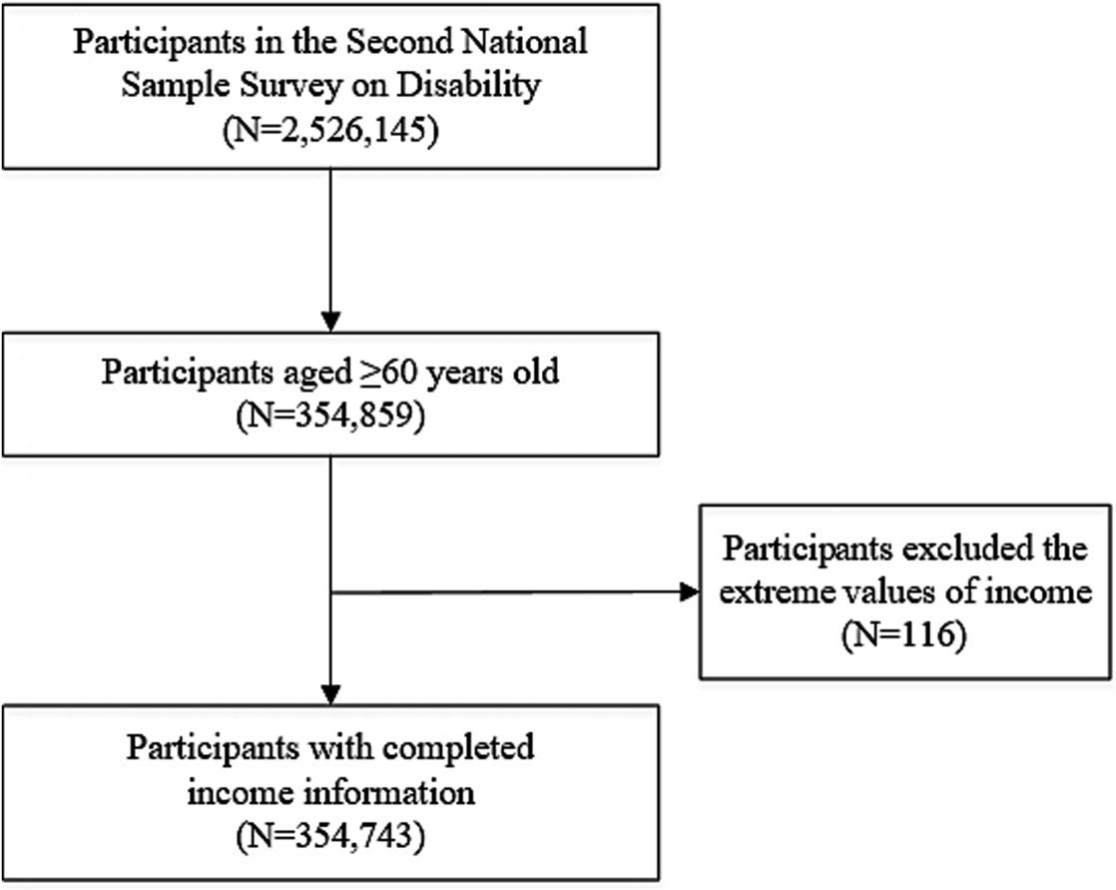
Flowchart of the study sample.

The number of participants in the CNSSD was 2,526,145. According to the Law of the People’s Republic of China on the Protection of the Rights and Interests of Older Adults, older adults referred to those over 60 years old; thus, we restricted our analysis to 354,895 adults. After excluding the extreme income values (equals 0 or 99,999, *n* = 116), we obtained a final study sample size of 354,743. The flowchart of the sample screening can be found in Figure 1.

### Measurement and variables

#### Cataract-induced visual disability

According to the Disability Standards of the CNSSD, visual disability refers to poor binocular vision or the constriction of the visual field caused for various reasons and is uncorrectable, affecting daily life and social participation (21). Visual disability can be divided into blindness and low vision. The survey adopted the screening questionnaire for persons with disabilities to conduct a household investigation and select suspected visual disabilities. In order to ensure the accuracy of screening, some quality control measures are taken. Investigators who conducted the door-to-door interviews were trained strictly. They were required to ask questions item by item, give the interviewees enough time to think, and pay attention to the behavior of all members of the household to identify possible omissions. They also had to make sure all family members present totally understand the questions and answered directly by themselves. Efforts also be made to improve the face-to-face meeting rate with the cooperation of the community workers. At the end of the investigation, the quality inspection team conducted several random checks on the screening quality of the investigators again, focusing on whether the screening existed omissions. For persons with suspected visual impairment, trained ophthalmologists with more than 5 years of clinical experience conducted a professional eye examination (21). Specialists performed vision examination using occlude, portable slit lamp, and other tools for a final diagnosis, then analyzed the cause of disability and provided advice on rehabilitation (21). If the pathogeny was diagnosed as a cataract, the case was categorized as “cataract-induced visual disability.” The definition and measurement of various types of disability are based on the International Classification of Functioning, Disability and Health (ICF) (21, 25), a classification system of disability and health officially promulgated by the World Health Organization in 2001, which is an important international taxonomy for classifying and measuring function, disability, and health with standardized concepts and terminology (26). The survey applied ICF’s theoretical pattern, classification and coding system to disability standard revision and survey scheme design. The grading standard of visual disability is shown in Table 1. Blind or low vision refers to both eyes. Active 2: In case of a difference in the degree of vision between both eyes, only the eye with better vision is considered. If only one eye is blind or has low vision and the other eye has a vision of 0.3 or better, it does not belong to visual disability. The BCVA refers to the best vision that can be achieved by appropriate lens rectification or vision measured by pinhole glass. Persons with visual field radius less than 10 degrees are recognized as visual disability, regardless of their vision.

**Table 1:** The grading standard of visual disability.

Category	Grade	Best corrected visual acuity (BCVA)*
Blindness	1	Visual acuity no light perception~ < 0.02; or visual field radius < 5 degrees
	2	Visual acuity 0.02 ~ < 0.05; or visual field radius < 10 degrees
Low vision	3	Visual acuity 0.05 ~ < 0.1
	4	Visual acuity 0.1 ~ < 0.3

Cited from Handbook on the main data of the second China national sample survey on disability.

*The visual acuity unit of measure is decimals.

#### Individual-level socioeconomic status

Individual-level SES was measured by household income, education, and occupation (27). We calculated household income *per capita* by summing all kinds of economic income for the household annually and dividing it by the number of household residents. It was treated as a continuous variable. In descriptive analyzes and single-factor test part, household income *per capita* was classified into “Low” “Middle” and “High” according to the tertiles. Education level was a five-response categorical variable, ranging from illiteracy to completion of undergraduate college or above, which was categorized by degree attainment. The occupation was divided into a dichotomous variable, referring to whether the respondent had engaged in paid social labor for at least 1 hour the week before the standard survey time.

#### Variables

The outcome variable in this study is whether an individual had a cataract-induced visual disability. The key independent variable is individual-level SES. Covariates include sex (male or female), age (5-year age groups, from 60 to 64 to ≥80), regional area (east, middle, west, or northeast), marital status (married or unmarried), household size (1, 2, 3, or ≥ 4) and residence (urban or rural).

### Statistical analysis

Firstly, we conducted descriptive analyzes to present the characteristics of the sample and the prevalence of cataract-induced visual disability in all older adults and in various demographic and socioeconomic groups. The chi-square test was applied to the single-factor test. Secondly, logistic regressions were used to analyze the associations between individual-level SES and cataract-induced visual disability. Model 1 concentrated on the whole sample. Model 2 and Model 3 considered rural participants and urban participants, respectively. Sampling weights were used to adjust for the complex sampling design of the CNSSD in all analyzes. To verify the robustness of the analyzes, sensitivity analyzes were performed. Statistical significance was set at a two-tailed *p*-value of <0.01. The reason we selected the significant *p*-value as <0.01 is that the conditions for rejecting the null hypothesis will be more stringent than <0.05. Therefore, the conclusions drawn on this basis will be more reliable. Stata 16.0 was used to conduct all statistical analyzes.

### Ethics approval

Approved by the State Council of China (No. 20051104), this survey was conducted in all provinces by the Leading Group of the CNSSD and the National Bureau of Statistics, according to legal guidelines governed by the Statistical Law of the People’s Republic of China. All survey respondents provided consent to the Chinese government.

## Results

### Characteristics of samples

Table 2 shows the characteristics of the study population. Among those with cataract-induced visual disability, females accounted for a larger scale than males. Individuals with cataract-induced visual disability were more concentrated in the aged ≥80 years and more likely to reside in east and rural areas. They also tended to be unmarried and live in a larger family (household size ≥4 people). In terms of individual-level SES, individuals with cataract-induced visual disability tended to have characteristics such as “low household income *per capita*” “illiteracy” and “unemployment.”

**Table 2:** Characteristics of participants.

	Sample N	Weighted N (%)	Visual disabilities caused by cataracts			*P* ^a^
			Sample	Weighted	Weighted prevalence	
			N	N	% (95% CI)	
Total	354,743	184,181,825	16,420	8,906,045	4.84 (4.76–4.91)	
Household income *per capita*						
Low	115,886	63,471,860 (34.46)	7,333	4,000,360	6.30 (6.16–6.45)	<0.01
Middle	117,584	64,216,045 (34.87)	5,822	3,254,229	5.07 (4.94–5.20)	
High	121,273	56,493,920 (30.67)	3,265	1,651,456	2.92 (2.82–3.03)	
Education level						
Illiteracy	157,945	85,130,049 (46.22)	11,592	6,342,337	7.45 (7.31–7.59)	<0.01
Primary school	119,167	62,736,368 (34.06)	3,802	2,051,063	3.27 (3.16–3.38)	
Junior high school	441 96	21,645,434 (11.75)	700	358,379	1.66 (1.53–1.79)	
Senior high school/ technical secondary school	21,499	9,879,090 (5.36)	231	112,518	1.14 (0–99-1.31)	
Undergraduate college and above	11,936	4,790,884 (2.60)	95	41,748	0.87 (0.70–1.09)	
Occupation						
Do not have	260 81	131,823,640 (71.57)	14,807	8,021,502	6.09 (5.99–6.19)	<0.01
Have	93,962	52,358,185 (28.43)	1,613	884,543	1.69 (1.61–1.78)	
Sex						
Female	182,892	94,901,014 (51.53)	10,796	5,873,171	6.19 (6.07–6.31)	<0.01
Male	171,851	89,280,811 (48.47)	5,624	3,032,874	3.40 (3.31–3.49)	
Age						
60–64	104,080	54,188,159 (29.42)	1,267	658,211	1.21 (1.15–1.29)	<0.01
65–69	89,259	46,169,280 (25.07)	2,253	1,182,201	2.56 (2.45–2.67)	
70–74	74,695	38,568,688 (20.94)	3,636	1,968,178	5.10 (4.94–5.27)	
75–79	48,386	25,105,101 (13.63)	3,934	2,152,486	8.57 (8.31–8.85)	
≥80	38,323	20,150,597 (10.94)	5,330	2,944,969	14. 61 (14.24–15.00)	
Area						
East	134,824	69,440,199 (37.70)	5,856	3,311,986	4.77 (4.64–4.90)	<0.01
Middle	82,488	49,502,243 (26.88)	3,942	2,400,373	4.85 (4.70–5.00)	
West	107,778	51,216,097 (27.81)	5,816	2,824,771	5.52 (5.37–5.66)	
Northeast	29,653	14,023,286 (7.61)	806	368,915	2.63 (2.45–2.82)	
Marital status						
Unmarried	13,652	59,666,136 (32.40)	9,004	4,912,976	8.23 (8.07–8.41)	<0.01
Married	241,091	124,515,689 (67.60)	7,416	3,993,069	3.21 (3.13–3.28)	
Residence						
Rural	231,898	127,876,880 (69.43)	12,581	6,997,252	5.47 (5.38–5.57)	<0.01
Urban	122,845	56,304,945 (30.57)	3,839	1,908,793	3.39 (3.28–3.50)	
Household size						
1	30,160	15,927,181 (8.65)	2068	1,149,652	7.22 (6.91–7.54)	<0.01
2	116,820	59,825,038 (32.48)	4,023	2,207,454	3.69 (3.57–3.81)	
3	52,690	27,029,473 (14.68)	2,218	1,203,308	4.45 (4.27–4.65)	
≥4	155,073	81,400,133 (44.20)	8,111	4,345,631	5.34 (5.22–5.46)	

^a^ Chi-square test for significant differences of categorical variables.

### Prevalence of cataract-induced visual disability

Table 2 demonstrates the prevalence of cataract-induced visual disability in the total study sample and different groups. The weighted estimates showed that 4.84% (95% CI: 4.76–4.91%) of the older adults in China suffered from cataract-induced visual disability in 2006. The prevalence among low, middle, and high *per capita* income indicated a stepwise pattern of decreasing disability prevalence by increasing income. A similar association was found between education level and disability prevalence. Older people without jobs had a much higher prevalence than those employed. Differences in prevalence were found in other dimensions; disability prevalence was higher in females, those in older age groups, western areas, unmarried people, rural residents and those who lived alone.

### The association between individual-level SES and cataract-induced visual disability

Table 3 reports logistic regression results of SES and cataract-induced visual disability. In Model 1, results revealed that a 1-logarithmic unit increase in household income *per capita* was associated with decreased risk of cataract-induced visual disability (OR = 0.83, 95% CI: 0.81–0.85, *p* < 0.01). As the education level improved, the disability risk declined gradually (reference group: illiteracy, primary school: OR = 0.80, 95% CI: 0.76–0.83, *p* < 0.01; junior high school: OR = 0.57, 95% CI: 0.52–0.62, *p* < 0.01; senior high school/technical secondary school: OR = 0.39, 95% CI: 0.33–0.45, *p* < 0.01; ≥undergraduate college: OR = 0.31, 95% CI: 0.25–0.39, *p* < 0.01). Having occupation significantly reduced the risk of cataract-induced visual disability (OR = 0.53, 95% CI: 0.50–0.56, *p* < 0.01). In the analysis of the subsamples, participants were divided into rural and urban residents, and regression outcomes were shown in Model 2 and Model 3. Estimates demonstrated that the increase in household income *per capita* and education level played a more significant role in decreasing disability risk in the urban areas, while having occupation contributed more to reducing risk in the rural areas.

**Table 3:** Logistic regressions of the association between SES and cataract-induced visual disability.

	Model 1 (Total)	Model 2 (Rural)	Model 3 (Urban)
	Odds ratio (95%CI)	Odds ratio (95%CI)	Odds ratio (95%CI)
Ln (Household income *per capita*)	0.83^***^ (0.81, 0.85)	0.86^***^ (0.84, 0.88)	0.74^***^ (0.71, 0.77)
Education level			
Illiteracy	Reference	Reference	Reference
Primary school	0.80^***^ (0.76, 0.83)	0.83^***^ (0.79, 0.87)	0.73^***^ (0.67, 0.80)
Junior high school	0.57^***^ (0.52, 0.62)	0.63^***^ (0.56, 0.71)	0.54^***^ (0.46, 0.62)
Senior high school/ technical secondary school	0.39^***^ (0.33, 0.45)	0.51^***^ (0.41, 0.64)	0.36^***^ (0.29, 0.44)
Undergraduate college and above	0.31^***^ (0.25, 0.39)	0.42^**^ (0.21, 0.84)	0.35^***^ (0.27, 0.45)
Occupation			
Do not have	Reference	Reference	Reference
Have	0.53^***^ (0.50, 0.56)	0.51^***^ (0.48, 0.54)	0.63^***^ (0.52, 0.76)
Sex			
Female	Reference	Reference	Reference
Male	0.78^***^ (0.75, 0.81)	0.80^***^ (0.76, 0.84)	0.67^***^ (0.61, 0.73)
Age			
60–64	Reference	Reference	Reference
65–69	1.78^***^ (1.65, 1.91)	1.79^***^ (1.64, 1.95)	1.76^***^ (1.51, 2.06)
70–74	3.03^***^ (2.82, 3.26)	3.07^***^ (2.83, 3.34)	2.98^***^ (2.57, 3.46)
75–79	4.73^***^ (4.39, 5.09)	4.75^***^ (4.36, 5.17)	4.81^***^ (4.12, 5.60)
≥80	7.75^***^ (7.19, 8.35)	7.57^***^ (6.95, 8.26)	8.65^***^ (7.42, 10.08)
Area			
East	Reference	Reference	Reference
Middle	0.98 (0.94, 1.03)	1.02 (0.97, 1.07)	0.86^***^ (0.78, 0.95)
West	1.21^***^ (1.16, 1.26)	1.25^***^ (1.19, 1.31)	1.09^**^ (1.00, 1.19)
Northeast	0.64^***^ (0.59, 0.69)	0.71^***^ (0.65, 0.78)	0.51^***^ (0.45, 0.59)
Marital status			
Unmarried	Reference	Reference	Reference
Married	0.85^***^ (0.81, 0.89)	0.84^***^ (0.80, 0.88)	0.92^*^ (0.84, 1.01)
Household size			
1	Reference	Reference	Reference
2	0.94^**^ (0.88, 1.00)	0.95 (0.88, 1.03)	0.88^*^ (0.77, 1.01)
3	0.93^**^ (0.87, 1.00)	0.95 (0.87, 1.02)	0.86^**^ (0.75, 0.99)
≥4	1.04 (0.98, 1.10)	1.05 (0.98, 1.12)	0.94 (0.84, 1.05)
Residence			
Rural	Reference	——	——
Urban	0.82^***^ (0.78, 0.86)	——	——

Exponentiated coefficients; 95%CI in parentheses.

^*^ *p* < 0.1, ^**^ *p* < 0.05, ^***^ *p* < 0.01.

To verify the robustness of our analyzes, sensitivity analyzes were performed, and outcomes were shown in the Supplementary materials. Firstly, we kept the samples with extreme income values and reran the models. The regression results remained largely consistent (see Supplementary Table S1). Furthermore, we adopted another comprehensive partitioning of individual-level SES. In this way, overall SES was measured by the sum of the z-score for each SES variable. The z-scores of individual-level SES ranged from-2.24 to 22.57, and a higher score means better individual-level SES. As shown in Supplementary Table S2, the estimation results were unchanged with this variable setting.

## Discussion

Our data, drawn from a national-wide representative survey, indicated that approximately 5 in every 100 older adults in China had cataract-induced visual disability in 2006. As far as we know, this is the first study to reveal the association between individual-level SES and cataract-induced visual disability among older adults based on a nationally representative sample in China. The findings of this study provided evidence about cataract-induced visual disability in China, which enriches perspectives for the promotion of eye health worldwide, especially in low-and middle-income countries.

Our results demonstrated a strong correlation between individual-level SES and the visual disability prevalence of cataracts after adjusting for confounding variables. Older people with higher household income, better education background, and occupation were less likely to suffer from cataract-induced visual disability. It comported well with previous studies that lower SES was associated with a worse health condition (1, 28, 29). Individuals with higher household incomes were more likely to live in better economic conditions and have higher life quality, which can reduce their exposure to the environmental risks of visual impairment, such as intense ultraviolet (UV), inferior screens, and so on. Cataract surgery is among the most cost-effective healthcare interventions, which can prevent further deterioration of vision impairment and avoid cataract-induced visual disability (1, 30, 31). However, not everyone has this opportunity (30, 32). High income gives individuals access to cataract surgery by providing an economic foundation. Several studies have suggested a correlation between lower education levels and higher risks for unhealthy lifestyles (e.g., long time for electronic products, unhealthy diets, and physical inactivity) and poor health consciousness related to eye health (33–35). Besides, people with a high level of education may work in upper-class occupations and enjoy a more comfortable work environment (36, 37). In this way, they can avoid the harm from outdoor UV exposure or strong-light stimulation in the factory, which are the risk factors of cataracts (24). Compared with unemployed people, those with jobs are naturally involved in more social participation and social network. They can gain more social capital, which increases their physical activity level and adjusts their mental health (38, 39). Work also makes people’s daily life schedules more regular and helps individuals to develop a disciplinary lifestyle. Good body condition and habits have been demonstrated to benefit eye health (40, 41). Concentrating on work may make people more sensitive to their visual abnormality, so they can intervene and correct visual impairment earlier to avoid further disability. Relatively higher occupational prestige and SES also correspond to higher awareness of eye health protection (29). Notably, from the life course perspective, there is a cumulative effect of SES on eye health. People who maintain advantaged/disadvantaged will have better/worse eye health with advancing age (42, 43). The influence of covariates was consistent with previous studies (2, 15, 24, 44, 45).

Furthermore, this study indicated the existence of different mechanisms between urban and rural areas on the correlations between SES and cataract-induced visual disability. The increases in household income *per capita* and education level were related to more sensitive variation in decreasing disability risk in urban areas, while having occupation contributed more to reducing the risk of disability in rural areas. China’s long-standing rural–urban dual structure has brought unbalanced regional development in allocating infrastructure resources, including healthcare accessibility, service cost, and welfare policies (46, 47). As eye care and cataract surgery were seriously insufficient in rural areas, the roles of material wealth and education were greatly limited, just like “the ceiling.” Even if individuals have money and good healthcare awareness in rural areas, they still face restrictions on access to medical resources, including cataract surgery. The shortage not only limits access to ophthalmic services but also leads to higher financial burdens for older adults in rural areas when seeking eye care services (48, 49), weakening the effect of wealth. Because of the differences in historical tradition, economic resources, pension systems, and so on, the living styles between rural and urban areas fell into two distinct modes. Rural residents usually make a living by cultivation and face the “ceaseless toil” situation. They rarely obey the labor rules and regulations of the company and “be their own bosses” in the field, so the legally statutory retirement age has little effect. In this case, rural older adults may decide whether to work primarily based on their health condition (50, 51). As a result, occupation in rural areas is an indicator more closely related to the individual’s physical functioning and mental health (51). Previous studies have found that health factors, especially mobility, are essential for the vision health of old people (17). Therefore, older people with occupations in rural areas were more likely to face a lower risk of cataract-induced visual disability than their counterparts in urban areas.

Several strengths were presented in this study. Firstly, this is the first study to investigate the relationship between individual-level SES and cataract-induced visual disability among the older Chinese population. And individuals were divided into rural and urban samples for further research on exploring different mechanisms, which provided evidence for more targeted measures to prevent cataract-induced disabilities. Secondly, the CNSSD’s definition of types and causes of disability was based on rigorous medical diagnoses and conducted by a team of professional doctors, instead of self-reported methods, which made the measurement of disability more robust. Thirdly, nationally representative data with a very large sample size secured a high degree of statistical power and more precise estimates for the correlation between individual-level SES and cataract-induced visual disability. The results of our study can promote the high-quality development of eye health care, advance the realization of the 14th Five-Year Plan (2021–2025) for National Eye Health of the People’s Republic of China, and also provide support for WHO integrated people-centered eye care and the SDGs.

Despite the strengths mentioned above, the present analysis has some limitations. Firstly, we could not obtain the specific categories of participants’ occupations for further classification due to data restrictions. Secondly, patients who have already undergone surgery would have normal vision and would not be detected during the screening phase. For example, urban areas have much better access to cataract surgery and have much larger scales of surgery performed, which would result in a lower visual disability prevalence of cataracts. In addition, self-reported way during the screening stage may miss some potential people with visual disability. It would also result in a lower prevalence of cataract-induced visual disability. But we believe the impact will be small and acceptable because the quality of the screening was ensured as much as possible. Future surveys and data should pay more attention to utilization of cataract surgery, which was important for understanding the causal relationship between SES and cataract-induced visual disability. Thirdly, some factors that can influence the disability prevalence of cataracts, such as diabetes, humidity, and UV radiation (24, 52) were not included in the present study because of the absence of information. Therefore, more high-quality data is needed to produce more robust estimates in the future. Finally, it has been a long time since the conduction of CNSSD in 2006. The timeliness of data has reduced to certain content. However, this survey is still the latest and largest survey aimed at the disabled in China, which has high data quality and good representatives so far. A new round of survey will be conducted in the near future, and this study can provide some evidence to support for the subsequent investigation.

Notwithstanding these limitations, the findings of this study showed the unequal situation of individual-level SES and visual disability prevalence of cataracts among older adults in China and presented different mechanisms between rural and urban areas. In consideration of the gap in individual-level SES and corresponding visual health inequities, developing system-wide actions to promote the individual-level SES among vulnerable groups and areas which face higher risks is the key point of national eye health improvement. We should pay more attention to low individual-level SES population, provide protection against the high risk of cataract-induced visual disability, and improve the disability reporting and rehabilitation system. Considering the unbalanced resource allocation between urban and rural areas, it is necessary to promote the coordinated development of urban and rural areas and improve the construction of rural infrastructures and health services. Strategies to promote coordinated healthcare development regionally, strengthen safeguards for disadvantaged groups, and increase public awareness of visual disability prevention are warranted.

## Data availability statement

The datasets analyzed during the current study are not publicly available due to legal restrictions, that is, the data contain potentially sensitive information. The State Council of China imposed the restrictions according to the Statistical Law of the People’s Republic of China (1996 Amendment). A de-identified minimal dataset of the quantitative data is available upon request to researchers who meet the criteria for confidential information, by sending a request to the Data Access Committee of Institute of Population Research, Peking University, No.5 Yi He Yuan Road, Beijing 100871, China (contact via e-mail at rkyjs@pku.edu.cn).

## Author contributions

YF: Investigation, Writing – original draft. SG: Investigation, Validation, Writing – review & editing. WD: Writing – review & editing. CC: Writing – review & editing. CZ: Writing – review & editing. XZ: Conceptualization, Funding acquisition, Writing – review & editing.
