# Supplementary material for: Individual-level socioeconomic status and cataract-induced visual disability among older adults in China: the overview and urban-rural difference
Source: Front Public Health. 2024 Jan 18;12:1289188. doi: 10.3389/fpubh.2024.1289188 (PMC10885563; doi:10.3389/fpubh.2024.1289188)
Supplement: Supplementary file 1 [file Table_1.DOCX]

**Supplementary Table 1 Logistic regressions of the association between SES and cataract-induced visual disability (including extreme values of income)**

|  | **Model 1 (Total)** | |  | **Model 2 (Rural)** | |  | **Model 3 (Urban)** | |
| --- | --- | --- | --- | --- | --- | --- | --- | --- |
|  | **Odds ratio (95%CI)** | |  | **Odds ratio (95%CI)** | |  | **Odds ratio (95%CI)** | |
| **Ln (Household income per capita+1)** | | 0.83^***^ (0.82,0.85) | | | 0.86^***^ (0.84,0.88) | | | 0.76^***^ (0.73,0.79) |
| **Education level** | |  | | |  | | |  |
| Illiteracy | | Reference | | | Reference | | | Reference |
| Primary school | | 0.80^***^ (0.76,0.83) | | | 0.83^***^ (0.79,0.87) | | | 0.72^***^ (0.66,0.79) |
| Junior high school | | 0.56^***^ (0.52,0.62) | | | 0.63^***^ (0.56,0.71) | | | 0.53^***^ (0.46,0.61) |
| Senior high school/  technical secondary school | | 0.38^***^ (0.33,0.44) | | | 0.51^***^ (0.41,0.64) | | | 0.35^***^ (0.29,0.43) |
| Undergraduate college and above | | 0.31^***^ (0.25,0.39) | | | 0.42^**^ (0.21,0.83) | | | 0.34^***^ (0.26,0.43) |
| **Occupation** | |  | | |  | | |  |
| Don’t have | | Reference | | | Reference | | | Reference |
| Have | | 0.53^***^ (0.50,0.56) | | | 0.51^***^ (0.48,0.54) | | | 0.63^***^ (0.52,0.77) |
| **Sex** | |  | | |  | | |  |
| Female | | Reference | | | Reference | | | Reference |
| Male | | 0.78^***^ (0.75,0.81) | | | 0.80^***^ (0.76,0.84) | | | 0.67^***^ (0.62,0.74) |
| **Age** | |  | | |  | | |  |
| 60-64 | | Reference | | | Reference | | | Reference |
| 65-69 | | 1.77^***^ (1.65,1.91) | | | 1.79^***^ (1.64,1.95) | | | 1.76^***^ (1.50,2.06) |
| 70-74 | | 3.03^***^ (2.82,3.25) | | | 3.07^***^ (2.83,3.34) | | | 2.97^***^ (2.56,3.45) |
| 75-79 | | 4.72^***^ (4.38,5.09) | | | 4.74^***^ (4.35,5.16) | | | 4.79^***^ (4.11,5.58) |
| ≥80 | | 7.74^***^ (7.18,8.34) | | | 7.56^***^ (6.94,8.24) | | | 8.63^***^ (7.40,10.06) |
| **Area** | |  | | |  | | |  |
| East | | Reference | | | Reference | | | Reference |
| Middle | | 0.99 (0.94,1.03) | | | 1.02 (0.97,1.07) | | | 0.87^***^ (0.79,0.96) |
| West | | 1.21^***^ (1.16,1.26) | | | 1.25^***^ (1.19,1.31) | | | 1.11^**^ (1.02,1.20) |
| Northeast | | 0.64^***^ (0.59,0.69) | | | 0.71^***^ (0.65,0.78) | | | 0.52^***^ (0.45,0.59) |
| **Marital status** | |  | | |  | | |  |
| Unmarried | | Reference | | | Reference | | | Reference |
| Married | | 0.85^***^ (0.81,0.89) | | | 0.84^***^ (0.80,0.88) | | | 0.92^*^ (0.84,1.01) |
| **Household size** | |  | | |  | | |  |
| 1 | | Reference | | | Reference | | | Reference |
| 2 | | 0.94^**^ (0.88,1.00) | | | 0.95 (0.88,1.03) | | | 0.88^*^ (0.77,1.01) |
| 3 | | 0.93^**^ (0.87,1.00) | | | 0.94 (0.87,1.02) | | | 0.86^**^ (0.75,0.99) |
| ≥4 | | 1.04 (0.98,1.10) | | | 1.05 (0.98,1.12) | | | 0.94 (0.84,1.06) |
| **Residence** | |  | | |  | | |  |
| Rural | | Reference | | | —— | | | —— |
| Urban | | 0.82^***^ (0.78,0.86) | | | —— | | | —— |

Exponentiated coefficients; 95%CI in parentheses

^*^ *p* < 0.1, ^**^ *p* < 0.05, ^***^ *p* < 0.01

Household income per capita included the minimum value (=0) and the maximum value (=99,999).

**Supplementary Table 2 Logistic regressions of the association between SES and cataract-induced visual disability (using comprehensive index of individual-level SES)**

|  | **Model 1 (Total)** | |  | **Model 2 (Rural)** | |  | **Model 3 (Urban)** | |
| --- | --- | --- | --- | --- | --- | --- | --- | --- |
|  | **Odds ratio (95%CI)** | |  | **Odds ratio (95%CI)** | |  | **Odds ratio (95%CI)** | |
| **Socioeconomic status** | | 0.75^***^ (0.74,0.77) | | | 0.76^***^ (0.74,0.77) | | | 0.74^***^ (0.72,0.77) |
| **Sex** | |  | | |  | | |  |
| Female | | Reference | | | Reference | | | Reference |
| Male | | 0.77^***^ (0.74,0.80) | | | 0.80^***^ (0.77,0.84) | | | 0.65^***^ (0.60,0.71) |
| **Age** | |  | | |  | | |  |
| 60-64 | | Reference | | | Reference | | | Reference |
| 65-69 | | 1.80^***^ (1.67,1.94) | | | 1.81^***^ (1.66,1.97) | | | 1.78^***^ (1.52,2.08) |
| 70-74 | | 3.10^***^ (2.89,3.33) | | | 3.13^***^ (2.88,3.40) | | | 3.04^***^ (2.63,3.53) |
| 75-79 | | 4.86^***^ (4.51,5.23) | | | 4.85^***^ (4.45,5.28) | | | 4.96^***^ (4.27,5.77) |
| ≥80 | | 7.96^***^ (7.39,8.57) | | | 7.74^***^ (7.10,8.43) | | | 8.94^***^ (7.70,10.38) |
| **Area** | |  | | |  | | |  |
| East | | Reference | | | Reference | | | Reference |
| Middle | | 0.98 (0.94,1.02) | | | 1.01 (0.96,1.07) | | | 0.85^***^ (0.77,0.94) |
| West | | 1.20^***^ (1.16,1.26) | | | 1.24^***^ (1.18,1.30) | | | 1.08^*^ (1.00,1.18) |
| Northeast | | 0.62^***^ (0.57,0.67) | | | 0.70^***^ (0.64,0.77) | | | 0.49^***^ (0.43,0.56) |
| **Marital status** | |  | | |  | | |  |
| Unmarried | | Reference | | | Reference | | | Reference |
| Married | | 0.86^***^ (0.82,0.90) | | | 0.85^***^ (0.81,0.89) | | | 0.92^*^ (0.84,1.01) |
| **Household size** | |  | | |  | | |  |
| 1 | | Reference | | | Reference | | | Reference |
| 2 | | 0.92^**^ (0.87,0.99) | | | 0.95 (0.88,1.02) | | | 0.86^**^ (0.76,0.99) |
| 3 | | 0.90^***^ (0.84,0.96) | | | 0.92^**^ (0.85,1.00) | | | 0.82^***^ (0.71,0.94) |
| ≥4 | | 0.99 (0.93,1.04) | | | 1.01 (0.95,1.08) | | | 0.89^**^ (0.79,0.99) |
| **Residence** | |  | | |  | | |  |
| Rural | | Reference | | | —— | | | —— |
| Urban | | 0.82^***^ (0.78,0.85) | | | —— | | | —— |

Exponentiated coefficients; 95%CI in parentheses

^*^ *p* < 0.1, ^**^ *p* < 0.05, ^***^ *p* < 0.01

**Supplementary Table 3 Employment rates by residence and age groups**

|  | **unweighted** | | |  | **weighted** | |
| --- | --- | --- | --- | --- | --- | --- |
|  | **Rural (%)** | | **Urban (%)** |  | **Rural (%)** | **Urban (%)** |
| **Age** |  |  | | |  |  |
| 60-64 | 63.59 | 15.29 | | | 64.92 | 17.18 |
| 65-69 | 44.07 | 8.13 | | | 45.16 | 9.26 |
| 70-74 | 23.07 | 3.97 | | | 23.66 | 4.62 |
| 75-79 | 10.33 | 1.99 | | | 10.47 | 2.37 |
| ≥80 | 2.63 | 0.82 | | | 2.59 | 0.88 |

**Supplementary Table 4 Employment rates by residence and sex**

|  | **unweighted** | | |  | **weighted** | |
| --- | --- | --- | --- | --- | --- | --- |
|  | **Rural (%)** | | **Urban (%)** |  | **Rural (%)** | **Urban (%)** |
| **Sex** |  |  | | |  |  |
| Female | 24.92 | 4.51 | | | 25.60 | 5.22 |
| Male | 48.45 | 11.09 | | | 49.07 | 12.62 |
